# Case Report: Bilateral globus pallidum internus DBS for treating tremor and dystonia in spinocerebellar ataxia 17: a thirteen-year follow-up

**DOI:** 10.3389/dyst.2023.11363

**Published:** 2023-06-30

**Authors:** Aparna Wagle Shukla, Shilpa Chitnis, Irene A. Malaty, Pam Zeilman

**Affiliations:** 1 Department of Neurology, Fixel Institute for Neurological Diseases, University of Florida, Gainesville, FL, United States; 2 UT Southwestern Medical Center, Dallas, TX, United States

**Keywords:** dystonia, tremor, deep brain stimulation, SCA17, globus pallidus

## Abstract

**Background:**

Spinocerebellar ataxia 17 (SCA17) is a rare autosomal dominant trinucleotide disorder. There are no effective therapies for addressing the clinical symptoms of SCA17.

**Case report:**

We describe a 46-year-old male who presented with symptoms of generalized dystonia and focal arm tremors manifesting during adolescence. He underwent bilateral globus pallidus (GPi) DBS surgery that led to notable improvements in dystonia and tremor symptoms, impacting his quality of life. At the time of surgery, he did not show cerebellar ataxia features; however, these began to manifest 2 years after DBS surgery. He subsequently underwent genetic testing that confirmed the SCA17 diagnosis. Currently, at 13 years of follow-up, although the ataxia has continued to worsen, DBS therapy has led to persistent improvements in dystonia, tremor, and many aspects of quality of life.

**Discussion:**

The current case indicates that DBS is a promising symptomatic therapy for dystonia and tremor in SCA17.

## Introduction

Spinocerebellar ataxia (SCA) 17 is a rare form of autosomal dominant cerebellar ataxia resulting from an abnormal CAG expansion of the TATA-binding protein gene. In addition to the core symptoms of progressive cerebellar ataxia, the clinical phenotype can include dementia, epilepsy, psychosis, Parkinsonism, dystonia, and chorea [1]. Currently, there are no effective treatments for clinical symptoms of SCA17. We report a case of SCA17 presenting with generalized dystonia and bilateral arm tremors who demonstrated long-term improvements with deep brain stimulation (DBS) targeted to bilateral globus pallidus (GPi).

## Case report

**FIGURE 1:**
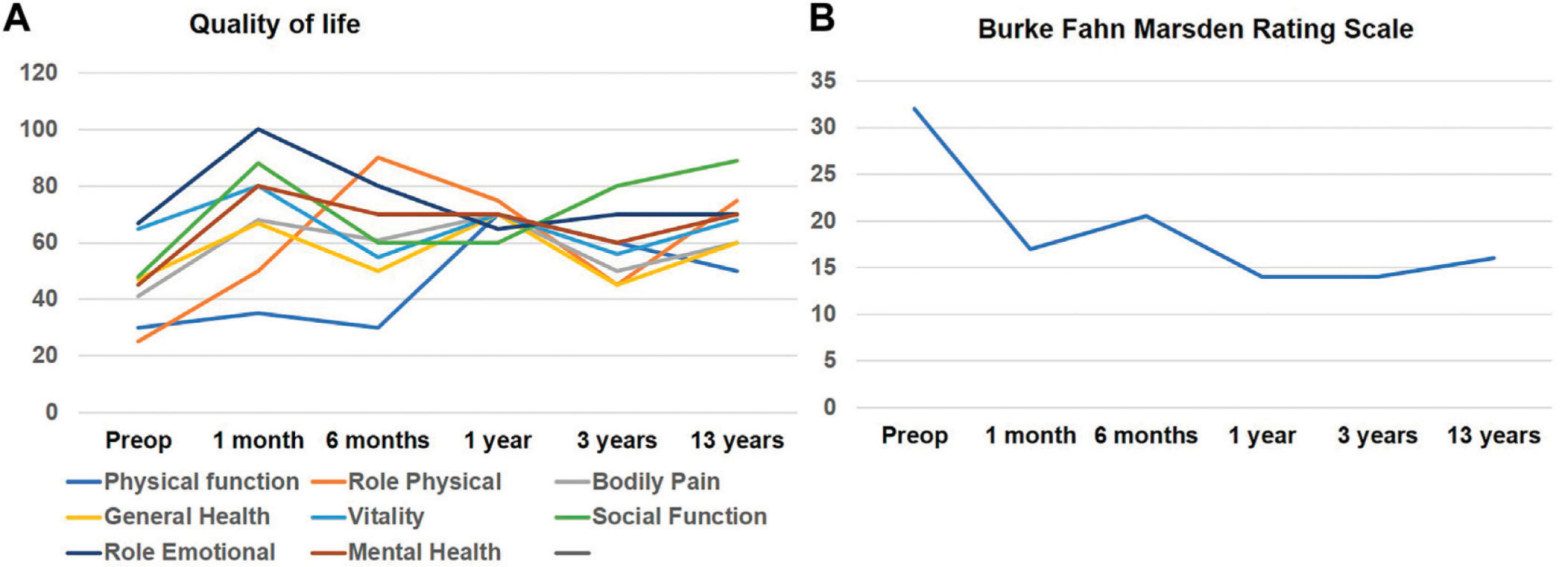
**(A)** illustrates line graphs of individual domain scores for SF-36 quality-of-life assessment that was recorded longitudinally after DBS surgery. Physical Function; Role physical and Social Function domains showed initial improvement which seemed to diminish over time however remained higher than scores before surgery. **(B)** illustrates the Burke-Fahn-Marsden dystonia rating scale assessed at multiple time intervals after DBS surgery.

**FIGURE 2:**
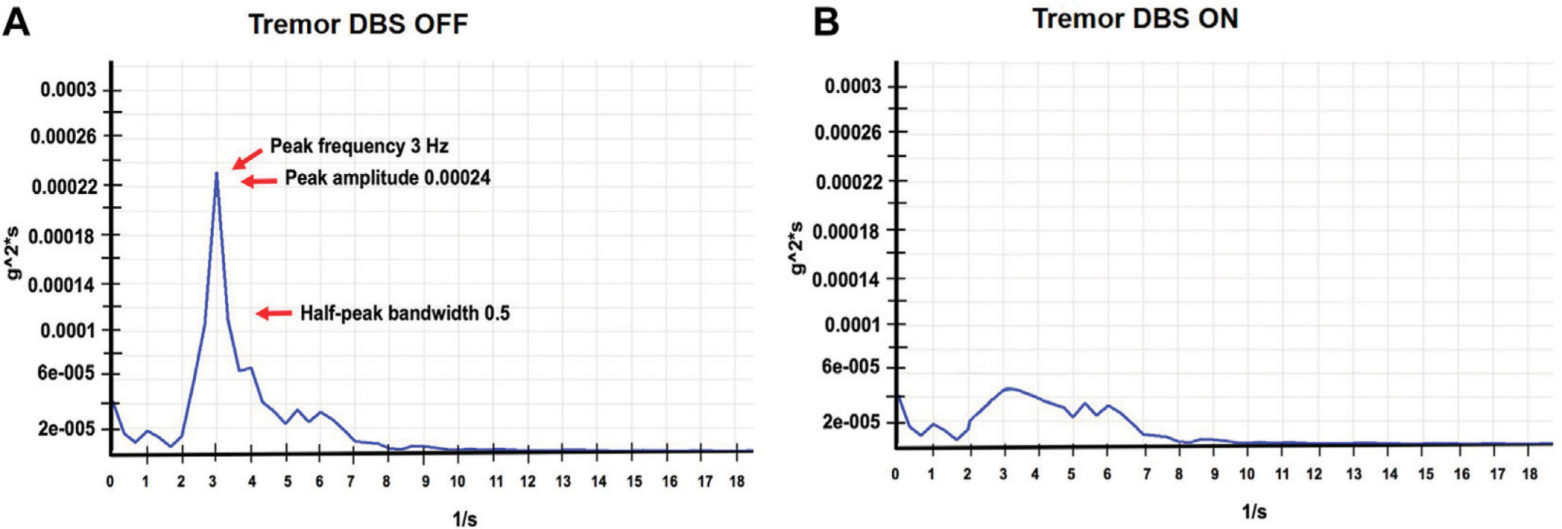
**(A,B)** represent accelerometer-based tremor recordings with DBS OFF, and DBS ON DBS (Left GPi, C+ 1-, 3V, 180 PW, 60 Hz; Right GPi, C+ 1-, 3V; 180 PW; 60 Hz), wash-out and wash-in intervals were 30 min respectively. These recordings were performed at the last follow-up visit. The figure illustrates the power spectrum analysis of the tremor signal. The raw signal was digitized, filtered (0–50 Hz) and was subjected to a fast fourier transform (FFT) analysis to generate the frequency peak. We first divided the selected data series (10 s) into overlapping sections of a specified window length, and window overlap and the squared FFT magnitude of each section was averaged and zero-padded to identify the dominant frequency peak. The tremor amplitude was calculated as a square root of the summated power of the frequency peaks recorded along the x, y, and z-axes. The width of the spectral peak at one-half the peak amplitude in the power spectrum was calculated to determine the cycle-to-cycle variability in the frequency (half peak bandwidth > 2 Hz indicates a more irregular tremor).

A 46-year-old right-handed white male presented to our center with adolescent-onset symptoms of painful posturing of the neck, arms, trunk, and toes. He had tremors affecting his arms that interfered with writing, eating, drinking, and dressing activities. With progression, he began to experience symptoms of chronic anxiety, depression, and panic attacks. He reported an awkward “lazy gait” but denied falls. Physical examination revealed that the neck was deviating to the left almost 30° and tilting to the right 20°, with some overall forward pulling. The wrist exhibited mild posturing, the trunk had right latero-flexion, the feet had plantar-flexion and inversion, and the toes (left > right) involuntarily flexed when walking. He had mild-moderate symmetric arm tremors, mainly kinetic, with minimal resting and no intentional components. There was no dysmetria/dysdiadochokinesis present on rapid repetitive movements. The spiral drawing task revealed a moderate amplitude jerky tremor with no axis. The remaining parts of the examination were unremarkable, including cognition, eye movement assessment, and motor system testing. His workup, such as MRI brain testing for Wilson’s disease and dystonia gene panel, was unremarkable. He received trials of trihexyphenidyl, levodopa-carbidopa, clonazepam, baclofen, and multiple rounds of botulinum toxin injections to the neck muscles with no improvements, deeming symptoms to be medication-refractory. Therefore, he underwent bilateral GPi DBS surgery to address the symptoms of dystonia and dystonic tremor. DBS leads were confirmed to be well placed postoperatively. The arm tremors responded soon after surgery; however, symptoms of dystonia required trials of wide monopolar and bipolar configurations at higher pulse widths of 450 μs and a range of frequencies (60 Hz–180 Hz). After 6 months of continued programming, he finally improved on the monopolar settings of 2 V amplitude, 120 μs pulse width, and 130 Hz frequency. The neck dystonia became less problematic, and he could ambulate more effectively in public spaces. Physical examination conducted at one and 6 months after DBS with the help of Burke Fahn Marsden rating scale, demonstrated improvements. The quality of life tracked with the SF-36 quality of life scale also revealed improvement in many domains (Figure 1). At the 1-year visit, he had some persistent difficulties with gait (Supplementary Video S1 reveals video collected with DBS turned off and on), despite reporting improvements in dystonia severity. About 2–3 years after surgery, he noticed significant impairments in speech and gait regardless of whether DBS was turned on or off (Supplementary Video S1 segment). Physical examination at this point revealed clear dysmetria in both arms and significant gait ataxia. These new features prompted a further workup, including serum levels for alpha-fetoprotein, albumin, amino acid, cholesterol, very long chain fatty acid, ammonia, transferrin factor, paraneoplastic antibodies, heavy metals, vitamin E, and ceruloplasmin. The urine was checked for elevated levels of organic acid, phytanic acid, frataxin, and lactic acid. Genetic testing was pursued to investigate a possible genetic form of ataxia, although the family history was negative. SCA panel revealed an unstable CAG trinucleotide expansion mutation coding for polyglutamine tracts in the TBP. There was one allele with 22 repeats and the other allele with 43 repeats. The findings of the testing were consistent with a diagnosis of SCA17. At 13 years of follow-up, even though ataxia symptoms progressed, the patient endorsed enduring improvements in dystonia and tremor with DBS turned on (Left GPi, C+ 1-, 3 V, 180 PW, 60 Hz; Right GPi, C+ 1-, 3 V; 180 PW; 60 Hz). Some DBS programming studies have found clinical benefits for dystonia when using low frequencies [2]. At this follow-up visit, we conducted an accelerometer-based electrophysiological testing of the tremor that revealed a 3 Hz low-frequency band with a slightly broad half-peak bandwidth. Importantly the tremor peak was observed to go away when the DBS was turned on (Figure 2). He reported that his activities of daily living were easier with DBS turned on due to effective tremor control. He found that the quality of life compared to before surgery was better in domains pertaining to social functions, and physical and mental health.

## Discussion

We report long-term outcomes of bilateral GPi DBS therapy in a patient with SCA17. Unlike previous SCA17 reports of focal dystonia (writer’s cramp and cervical dystonia) [3], the current case presented with dystonia generalized in distribution. The cerebellar ataxia symptoms manifested two decades after the initial symptoms, highlighting the wide variability in the phenotypic spectrum and disease course reported in the literature. While the CAG repeats cut-off for symptomatic manifestation is 43, many recent publications have reported clinical symptoms even with lower repeat expansion numbers [4]. Literature has only a few cases that reported DBS outcomes for genetic ataxia, such as SCA1, SCA 2, and SCA3. In recent series of SCA3 patients, the dentate nucleus of the cerebellum was targeted with DBS to improve ataxia symptoms. DBS was observed to be safe and well tolerated but did not improve ataxia symptoms [5]. With regards to dystonia, one report of SCA1 demonstrated partial benefit [6] In another report of patients with SCA2 and SCA3, there was some improvement in dystonia with bilateral GPi stimulation [7]. DBS outcome for the current case was earlier reported at a 1-year follow-up [8]. Although the patient continued to improve for another year, he later began to report clinical worsening mainly related to the emergence of ataxia over 20 years into his disease. DBS improved arm tremors, maintained at 13 years of follow-up with electrophysiological assessment revealing the tremor peak to suppress in response to DBS turning on. While the diagnosis for SCA17 was indeed delayed, our current case demonstrated that DBS could lead to long-term symptom-specific benefits, accompanied by improvement in the activities of daily living and quality of life. Whether cerebellar ataxia was unmasked with improvement in tremor and dystonia or DBS therapy acted as a trigger for delayed presentation of ataxia symptoms a few years later is unclear.

Our case had some additional unique features. Unlike previous cases that selected the thalamus to control tremors [9], our patient with a tremor in the setting of dystonia revealed benefits with GPi stimulation. None of the previous cases reports or case series reported electrophysiological characterization of tremor in SCA17 or has provided data on long-term follow-up. We recognize that the symptomatic improvement seen in our case of SCA17 cannot be generalized to all other forms of inherited ataxias. Our case presentation does not include video recordings for all time points. More cases with blinded assessments will be needed. We recognize that DBS cannot address all features of an ataxia syndrome. Nevertheless, DBS in SCA17 has shown a promising potential to address specific extrapyramidal features such as tremors and dystonia.

## Supplementary Material

Video

## Data Availability

The raw data supporting the conclusion of this article will be made available by the authors, without undue reservation.
